# Identification of ∆9-tetrahydrocannabinol (THC) impairment using functional brain imaging

**DOI:** 10.1038/s41386-021-01259-0

**Published:** 2022-01-08

**Authors:** Jodi M. Gilman, William A. Schmitt, Kevin Potter, Brian Kendzior, Gladys N. Pachas, Sarah Hickey, Meena Makary, Marilyn A. Huestis, A. Eden Evins

**Affiliations:** 1 Massachusetts General Hospital (MGH) Department of Psychiatry, Boston, MA USA; 2 Harvard Medical School, Boston, MA USA; 3 MGH/HST Athinoula A. Martinos Center for Biomedical Imaging, Department of Radiology, Massachusetts General Hospital, Harvard Medical School, Charlestown, MA USA; 4 Faculty of Engineering, Cairo University, Cairo, Egypt; 5 Institute of Emerging Health Professions, Thomas Jefferson University, Philadelphia, PA USA

**Keywords:** Neuroscience, Predictive markers

## Abstract

The primary cannabinoid in cannabis, Δ9-tetrahydrocannabinol (THC), causes intoxication and impaired function, with implications for traffic, workplace, and other situational safety risks. There are currently no evidence-based methods to detect cannabis-impaired driving, and current field sobriety tests with gold-standard, drug recognition evaluations are resource-intensive and may be prone to bias. This study evaluated the capability of a simple, portable imaging method to accurately detect individuals with THC impairment. In this double-blind, randomized, cross-over study, 169 cannabis users, aged 18–55 years, underwent functional near-infrared spectroscopy (fNIRS) before and after receiving oral THC and placebo, at study visits one week apart. Impairment was defined by convergent classification by consensus clinical ratings and an algorithm based on post-dose tachycardia and self-rated “high.” Our primary outcome, prefrontal cortex (PFC) oxygenated hemoglobin concentration (HbO), was increased after THC only in participants operationalized as impaired, independent of THC dose. ML models using fNIRS time course features and connectivity matrices identified impairment with 76.4% accuracy, 69.8% positive predictive value (PPV), and 10% false-positive rate using convergent classification as ground truth, which exceeded Drug Recognition Evaluator-conducted expanded field sobriety examination (67.8% accuracy, 35.4% PPV, and 35.4% false-positive rate). These findings demonstrate that PFC response activation patterns and connectivity produce a neural signature of impairment, and that PFC signal, measured with fNIRS, can be used as a sole input to ML models to objectively determine impairment from THC intoxication at the individual level. Future work is warranted to determine the specificity of this classifier to acute THC impairment.

ClinicalTrials.gov Identifier: NCT03655717

## Introduction

Intoxication with Δ9-tetrahydrocannabinol (THC), the psychoactive ingredient in cannabis, impairs cognitive and psychomotor performance, impairs driving, and at least doubles the risk of fatal motor vehicle crashes [1]. A pre-specified THC metabolite concentration in body fluids as a proxy for intoxication or impairment is still used in many parts of the US, analogous to blood alcohol concentration limits, but this is prone to false-positive results. THC metabolites can remain in the bloodstream for weeks after last use, long after the period of intoxication is over [2] and does not correlate well with impairment [3]. Accordingly, THC or THC metabolite concentrations in the breath or body fluids are unlikely to yield an accurate, reliable test of impairment [2, 4, 5]. In the absence of an accurate biometric, impairment due to cannabis intoxication has been measured and defined legally in many places using an enhanced field sobriety test (eFST) during traffic stops [6]. However, the eFST has been reported to be insensitive to oral THC [7] and prone to false positive bias [8]. The need for an objective, reliable method to detect impairment due to THC is well recognized in view of the absence of a reliable, objective, quantitative, biological test for impairment due to acute cannabis intoxication [1, 9, 10]. The present study assesses a brain-based method for determining impairment as an alternative to eFSTs.

Neural states of impairment from intoxicating substances, including cannabis, are poorly understood. As cannabinoid 1 (CB_1_) receptors, the main target of Δ9-THC, the primary intoxicating cannabinoid in cannabis, are densely localized within the fronto-limbic circuit [11], prefrontal brain regions are key locations to examine brain changes that characterize impaired clinical states associated with acute intoxication. THC increases dopamine release via activation of presynaptic CB1 receptors in the ventral tegmental area, via GABAergic and glutamatergic terminals [12], resulting in less functional connectivity in the mesocorticolimbic circuit [13] and increased connectivity (via increased glutamatergic signaling) in the prefrontal cortex [14, 15]. Indeed, neuroimaging studies have shown that THC exposure activates fronto-striatal reward circuitry, including the medial prefrontal cortex (PFC) [3, 16], and acute administration of THC increases perfusion (as assessed with arterial spin labeling) in prefrontal areas [17].

Functional near-infrared spectroscopy (fNIRS), a noninvasive and inexpensive method for assessing oxygenated hemoglobin (HbO) response, can be easily used to query the PFC. We previously reported that activation-induced changes in cerebral HbO concentration were significantly altered by THC intoxication [18]. Participants who reported intoxication after oral THC, compared with those reporting low or no intoxication after oral THC, had greater fNIRS-detected prefrontal cortical (PFC) oxygenated HbO response during an n-back working memory task [18, 19], suggesting that a neural activity signature, detectable with portable brain imaging, may characterize THC intoxication. To date, neuroimaging studies of the effect of THC on brain function have focused on THC exposure rather than impairment associated with acute intoxication from THC. Further, neuroimaging studies have compared group-level differences in brain states (e.g. THC vs placebo), with no study to date examining individual brain scans to determine impairment.

**Fig. 1:**
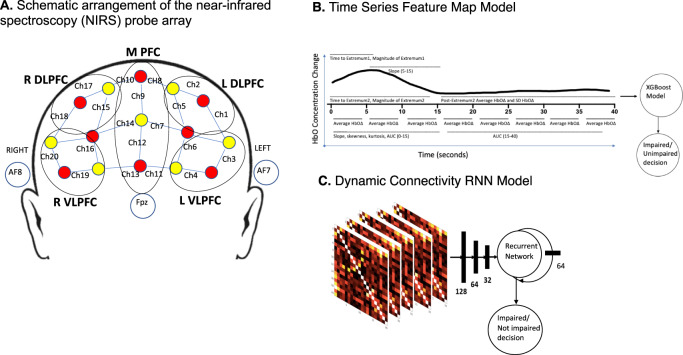
Design of probe and machine learning models. **A** The continuous wave near-infrared spectroscopy (NIRS) machine was used to measure changes in oxygenated hemoglobin (HbO) and deoxygenated hemoglobin (deoxy-Hb). The NIRS probe comprised of eight sources (red) and seven detectors (yellow) placed over the prefrontal brain region (forehead) of each participant. The mid-column of the probe was placed over Fpz, with the lowest probes located along the F5-Fp1-Fpz-Fp2-F6 line, in accordance with the International 10–20 Placement System. The distance between pairs of source and detector probes ranged from 2.5 - 3 cm. The midpoint of the source-detector distance was defined as channel (Ch) location, labeled numerically (1–20) in the above schematic. The channels were grouped into regions of interest, as illustrated in the schematic. We defined five regions on interest (ROIs) based on channel location. These ROIs are middle prefrontal cortex (MPFC, channels 7, 8, 9, 10, 11, 12, 13, 14); right dorsolateral prefrontal cortex (RDLPFC, channels 15, 17, 18); right ventrolateral prefrontal cortex (RVLPFC, channels 16, 19, 20); left dorsolateral prefrontal cortex (LDLPFC, channels 1, 2, 5); and left ventrolateral prefrontal cortex (LVLPFC, channels 3, 4, 6). **B** We extracted 95 numerical values (19 from each of the 5 ROIs): mean HbO values for time segments 0–5, 5–10, 10–15, 15–20, 20–25, 25–30, 30–35, and 35–40 s, slope (5–15 s), skewness (0–15 s), kurtosis (0–15 s), area under the curve (0–15, 15–40 s), time to first and second extremum of HbO, magnitude of first and second extremum of HbO, and average and standard deviation of HbO after the first extremum. We used these 95 predictive features to train XGBoost to predict impairment. **C** We computed pairwise correlations between the HbO concentration change values of all possible channel pairs. We computed dynamic connectivity matrices with sliding windows of 300 time points, and a skip of 100 time points, so that each window corresponded to a small segment of the scan. We then trained an RNN model architecture with the sliding-window correlation matrix feature vector as the input. In this RNN, the core component consisted of fully connected layers that mapped the input to a latent representation, which in turn fed to a hidden state with recurrent connections. A probabilistic prediction was computed at every time point by applying a fully connected layer to the hidden state. We used tanh for all nonlinearities and implemented a fully connected neural network with 5 hidden layers and 128, 64, 32, and 16 nodes. The output of this model was a fully connected neural net with a single hidden layer of 64 dimensions and consisted of recurrent connections that captured temporal dynamics.

Here, we aimed to better understand the brain state of impairment due to THC intoxication rather than the effect of THC exposure only and to use individual-level data and standard machine learning (ML) techniques to develop a diagnostic classification tool for THC impairment. To do so, we conducted a double-blind, cross-over study, randomized for order, of the effect of a single, individualized dose of synthetic THC designed to produce intoxication and identical placebo, approximately 1 week apart, in regular cannabis users, on PFC hemodynamics during the N-back working memory task using a 20-channel fNIRS probe (Fig. 1A) with one fNIRS scan before and two fNIRS scans following study drug at each study visit. A temporal feature-based ML model [20] (Fig. 1B) and a recurrent neural network (RNN) connectivity ML model [21] (Fig. 1C) were developed and used both separately and combined to classify individual participants as impaired or not clearly impaired from THC. Ground truth impairment, for the purpose of identifying fNIRS scans to be used in building the ML models, was operationalized as post-dose scans conducted when participants were (1) rated as impaired by two clinical raters, using all but fNIRS data, and (2) identified by an algorithm using physiologic (heart rate) and psychologic (self-rating of intoxication) inputs to discriminate those impaired following THC from not impaired following THC and placebo with a low false positive rate (FPR).

## Materials and methods

### Experimental design

Study procedures were approved by the Partners Human Subjects Committee. All participants provided written informed consent prior to initiation of study procedures. Participants were compensated for completion of each study visit. Recruitment started in January 2017 and was completed in March 2020.

### Participants

Adults, aged 18–55 years, who reported at least weekly cannabis use in the past 90 days were recruited through advertising in the community in the greater Boston area. Exclusion criteria included a negative urine THC (THC-COOH) screen (20 ng/mL cutoff; Medimpex United Inc., Bensalem, PA, USA), serious medical illness, lifetime history of schizophrenia spectrum or bipolar disorder, current regular use of benzodiazepines or barbiturates, antihistamines, atropine, scopolamine, or other anticholinergic agents, and known allergy to dronabinol or its constituents.

### Interventions

Participants were randomly assigned for order to receive a single oral dose of dronabinol (Marinol) capsules, an FDA-approved, synthetic THC, and single dose of identical appearing placebo capsules, on separate study visits, conducted at least 7 days apart (mean days apart = 9.3; SD = 15.5). Dronabinol dose was individualized with the goal of producing intoxication at a dose that was well tolerated by each participant up to a maximum of 80 mg, a dose that when given three times per day has been reported to be safe and generally well tolerated [22]. Dose was determined by taking a history of participants’ usual use pattern and estimated dose when used recreationally, and the decision to allow up to 80 mg dose was chosen to accommodate doses reported by participants while safely producing intoxication. Participants provided a detailed history of cannabis use pattern, approximate dose, route of administration, level of intoxication and adverse effects with various doses. Study staff used this information together with factors including sex and BMI to dose dronabinol for the study with the aim of maximizing the likelihood for intoxication for each individual participant while minimizing adverse effects such as nausea, anxiety, and hemodynamic change (see Supplementary Methods).

### Assessments

At the screening visit, participants provided a urine sample for quantitative analysis of 11-nor-9-carboxy-tetrahydrocannabinol (THCCOOH) concentration, the primary inactive THC metabolite, in order to exclude participants without recent cannabis use. Urine THCCOOH and creatinine concentrations were determined by liquid chromatography/tandem mass spectrometry, with the THCCOOH concentration normalized to the creatinine concentration (Dominion Diagnostics, Kingstown, RI, USA) [23]. Participants were asked to use no intoxicating substances the morning of the study visit. A qualitative urine drug screen was performed at screening and on each study day assessing for the presence of cannabinoids, opioids, cocaine, and amphetamines, in order to reschedule study visits for those with positive screens for opioids, cocaine, amphetamines. Participants were assessed at the beginning of each study visit. Those who arrived for a study visit with clinical signs of intoxication were rescheduled.

#### Intoxication

Subjective ratings of intoxication were collected at study visits before and at approximately 20-min intervals for approximately 240 min after study drug administration with the Drug Effects Questionnaire (DEQ; [24]), which consists of five questions assessing subjective drug effects, in which participants rated answers from 0 (no effects) to 100 (maximum effects).

#### Physiology

Heart rate (beats per minute; bpm) was measured before and approximately every 20 min for 240 min after THC/placebo administration.

#### fNIRS

Participants performed the n-back task during three fNIRS scans on each of the two study days; the first before THC/placebo administration, the second at approximately 100 min after THC/placebo administration, which corresponded to the estimated median maximum THC concentration in blood (Solvay Pharmaceuticals, 2004), and the third at approximately 200 min after THC/placebo administration. Each fNIRS scan consisted of a 6-minute run of the 0-back and 2-back condition of the letter n-back working memory (WM) task (six 30-s blocks, alternating 2-back, and 0-back) [18]. All participants practiced the n-back task at each visit before THC dosing and were given feedback on their performance. Reported analyses on behavioral task performance and fNIRS time-series scan data include the 2-back condition only.

#### Extended Field Sobriety Test

Immediately following the second fNIRS scan, approximately 120 min after THC/placebo dosing, a police officer who was trained and certified as a Drug Recognition Examiner (DRE), conducted the structured, extended field sobriety test used during traffic stops for suspected drugged driving, as described in the Advanced Roadside Impaired Driving Enforcement manual [6]. This test included all standardized assessments in the structured, extended field sobriety test except questioning of drugs used, including horizontal gaze nystagmus, pupillary response, walk and turn tests, and one leg stand (balance phase and counting phase), conducted in the specified order, and took approximately 45–60 min to perform.

#### Defining impairment

As there is no accepted objective definition of impairment, and because of the reported risk of high false-positive rates with the eFST [8], replicated in this study with over 21% of participants considered by the DRE to be impaired on the eFST following receipt of placebo, we developed a two-stage process for operationalizing ground truth impairment for the purpose of identifying fNIRS scans to be used in building the ML models. Post-dose scans were considered to be impaired if they were conducted when participants were rated as impaired by two clinical raters, using all but fNIRS data, in participants who were also identified by an algorithm using physiologic (heart rate) and psychologic (self-rating of intoxication) inputs to discriminate THC from placebo with a low FPR (see Supplementary Methods).

### Acquisition of fNIRS imaging data

A continuous wave-NIRS (NIRSport 8-8, NIRx, Medical Technologies LLC, Glen Head, NY, USA) device simultaneously acquired dual-wavelength (760 and 850 nm) near-infrared light to calculate relative concentration changes in oxygenated and deoxygenated hemoglobin (HbO and HbR, respectively) [25] based on the modified Beer-Lambert law [26]. The sampling frequency was 7.81 Hz. NIRStar software by NIRx verified the signal quality before each recording. NIRS data event markers were displayed, recorded and stored on the recording computer. The NIRS probe comprised eight sources and seven detectors placed over the PFC brain region of each participant (see Fig. 1A for a schematic). The mid-column of the probe was placed over Fpz, with the lowest probes located along the F5-Fp1-Fpz-Fp2-F6 line, in accordance with the International 10-20 Placement System [27]. The center of the cap was placed over the vertex (Cz) of each participant, at a point equidistant from both nasion (Nz) and inion (Iz) and equidistant from the left and right preauricular points. The distance between pairs of source and detector probes ranged from 2.5 to 3 cm. The midpoint of the source-detector distance was defined as channel (Ch) location.

### Statistical analysis

#### Analysis of fNIRS data

Our primary outcome measure in the study was HbO concentration. fNIRS analyses were conducted using Homer2 open source software (MGH-Martinos Center for Biomedical Imaging, Boston, MA, USA), implemented in MATLAB (Mathworks, Natick, MA, USA) [28]; see Supplementary Methods for detail. We defined five regions on interest (ROIs) based on channel location. See Fig. 1A.

#### Machine learning methods

Pre-processed data from impaired and placebo scans were used to build two models; a temporal feature map model from time-series data [29] using XGBoost (https://xgboost.readthedocs.io/en/latest/), an open-source distributed gradient boosting library that is normally used to train gradient-boosted decision trees and other models, and a RNN model from connectivity data [30]. See Supplementary Methods for detail. Ensemble learning combined the results of the XGBoost and RNN model architectures. We utilized boosting to iteratively fit the RNN model and use the classifier’s predictive results in combination with the extracted time-series features to fit the XGBoost model.

#### Cross validation and model construction (Temporal feature maps and RNN)

We constructed and examined all models with repeated five-fold cross-validation (five repeats), which partitioned the original sample into five subsets. Four subsets were part of the training process, and predictions were made for the remaining subset. Stratified k-fold validation ensured that each subset had an equal distribution of impaired/non-impaired scans. To avoid opportune data splits, we averaged model performance metrics across test folds and selected the best performing models by examining FPR divided by true negative rate.

We measured the significance of the model’s accuracy with a one-tailed binomial test of model accuracy relative to scrambled data (null-information rate). We also measured other relevant descriptions of model discrimination—including sensitivity, specificity, and area under curve (AUC)—at each stage.

#### Test (hold-out) dataset

The classifier above was built with scans from 80 impaired participants. As a test set, scans from the 57 participants who were given THC but determined by concordant consensus clinical rating and HR/self-rated high algorithm impairment determination to be “not clearly impaired” were used to test the classifier. Methods to test these scans were identical to the methods presented above.

## Results

One hundred sixty-nine participants (86 males, 83 females, mean age 25.2 ± 6.4 years) initiated a study visit at which they received study drug and had at least one post-drug fNIRS scan (see Table 1, Supplementary Figure 1). Participants who completed a placebo study visit but not a THC study visit were excluded from the analyses.

**Table 1:** Characteristics of study participants overall and by analysis group.

Variables	Overall	Impaired post active study drug (THC)	Not clearly impaired post active study drug (THC)	Discordant/No valid scans
Sample size	169	80	57	32
*Demographics*				
Age	25.2 (6.4)	24.4 (5.3)	26.3 (7.5)	25.4 (6.6)
Sex; % Male (*n*)	50.9% (86)	57.5% (46)	47.4% (27)	40.6% (13)
Race				
% White (*n*)	67.5% (114)	68.8% (55)	70.2% (40)	59.4% (19)
% Black (*n*)	11.2% (19)	5% (4)	14% (8)	21.9% (7)
% Asian (*n*)	6.5% (11)	10% (8)	3.5% (2)	3.1% (1)
% Multi-racial (*n*)	7.7% (13)	10% (8)	5.3% (3)	6.2% (2)
% Other (*n*)	7.1% (12)	6.2% (5)	7% (4)	9.4% (3)
Ethnicity; % Hispanic (*n*)	20.1% (34)	25% (20)	17.5% (10)	12.5% (4)
Years of education completed	15.3 (2.1)	15.4 (2.2)	15.3 (2.1)	15 (1.8)
*Cannabis use characteristics*				
Age began regular use^a^	19 (3.9)	18.6 (3.6)	19.7 (4.6)	18.8 (3.6)
Weekly users; % Yes (*n*)	42% (71)	48.8% (39)	38.6% (22)	31.2% (10)
Daily users; % Yes (*n*)	56.2% (95)	51.2% (41)	56.1% (32)	68.8% (22)
Used multiple times per day; % Yes (*n*)	45% (76)	36.2% (29)	56.1% (32)	46.9% (15)
Urine THC-COOH (ng/mL)	221.8 (473.2)	98.1 (147.5)	456.2 (795.3)	163 (136.1)
CUDIT score	12 (5.3)	11.6 (5.2)	12.4 (5.2)	12.4 (5.7)
*Psychiatric characteristics*				
STAI - State (Baseline)	31.9 (6)	31.2 (5.1)	32.2 (6.2)	33 (7.7)
Lifetime depression				
% Diagnosed (*n*)	17.2% (29)	12.5% (10)	17.5% (10)	28.1% (9)
Lifetime anxiety				
% Diagnosed (*n*)	18.9% (32)	15% (12)	22.8% (13)	21.9% (7)

Unless otherwise noted, values are means with standard deviations in parentheses.

^a^Self-report of approximate first age of at least monthly cannabis use.

**Fig. 2:**
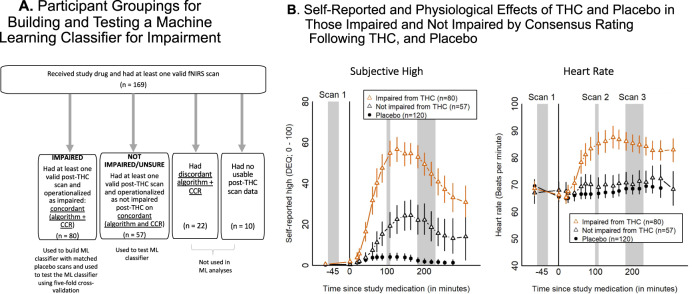
Psychological and physiological measures of participants by impairment and drug status. **A** Participants were assessed for impairment via an algorithmic approach and a clinical approach into “Impaired” and “Not Clearly Impaired” groupings, which were then used to build and test a classifier for impairment using fNIRS data. **B** Time course of **A** self-reported high (0–100 scale to answer the question: “Are you high right now?”, 0 being “Not at all” and 100 being “Extremely”) and **B** heart rate (beats per minute) were averaged over (1) individuals identified as impaired post-THC by consensus ratings (red triangles), (2) individuals identified as not clearly impaired post-THC by concordant ratings (black triangles), and (3) all individuals post-placebo (filled circles). Impairment status post-THC was determined based on concordant ratings between two rating methods, algorithmic and clinical experts. Error bars are 95% confidence intervals for the mean generated via bootstrap. Gray bars show time windows for the pre-dose (Scan 1) and post-dose (Scans 2 and 3) fNIRS imaging.

The clinical consensus ratings (CCR) classified 96 participants as impaired during a scan, and the HR/self-rated high algorithm classified 93 participants as intoxicated during a post-dose scan; 80 participants had scans with concordant CCR and algorithm ratings of impaired/intoxicated and were operationalized as impaired for building the ML classifier (Fig. 2A). The mean THC dose for these 80 participants considered to be impaired was 35.6 ± 11.5 mg. Likewise, 57 participants had concordant ratings of not clearly impaired on the CCR and HR/self-rated high algorithm (Fig. 2A); the mean dose of THC for these 57 participants rated as not clearly impaired was 34.8 ± 16.1 mg. The 80 participants operationalized as impaired post-THC had greater subjective, physiologic and cognitive (N-back performance) effects of THC than the 57 participants operationalized as not clearly impaired after receiving doses of THC that were not significantly different (Fig. 2B, Supplementary Table 1)*.* On the n-back task, d’ score (post-dose minus pre-dose) indicated that impaired participants showed a worsening in performance (d’ change = −0.22 ± 0.75), but participants who were not clearly impaired improved, likely benefiting from practice (d’ change = 0.21 ± 0.57), *t* = 3.70, *p* < 0.001.

There was 75.5% concordance between the CCR of impairment and algorithmic determination of impairment (Supplementary Fig. 2). Only post-THC scans with concordant impairment determinations and post-placebo scans (*n* = 80; see Fig. 2A) were used as ground truth to build the ML classifier of impairment. Of these 80 impaired participants, 39 were impaired at Scan 2 (100 min post-dose) only, 20 were impaired at Scan 3 (200 min post-dose) only, and 24 were impaired at both scans.

### Extended field sobriety test (eFST)

One hundred ten participants had a DRE-administered eFST after active THC, and 71 (64.5%) of these were impaired according to the eFST. Ninety-six participants had an eFST following placebo, and 21 (21.6%) of these were impaired according to the eFST. Of the participants who were operationalized as impaired (concordant CCR and algorithm ratings), 82.8% were also determined to be impaired by the eFST. Of the participants operationalized by concordant ratings as not clearly impaired post-THC, 60.9% were also determined to be not impaired by the eFST. THC dose did not differ significantly between participants determined to be impaired versus not impaired by the DRE-administered eFST (“eFST impaired” THC dose = 36.0 mg, “eFST not impaired” THC dose = 32.9 mg, *p* = 0.28).

### Group level effects of THC impairment on brain activation

**Fig. 3:**
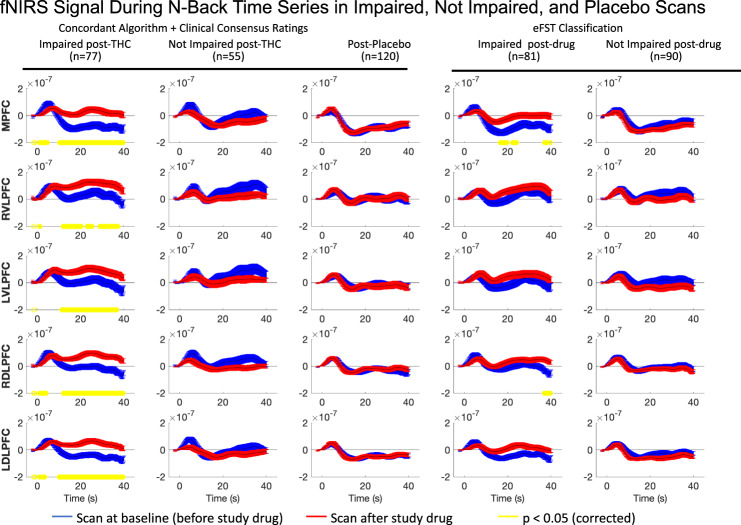
HbO response at pre- and post-dose by consensus rating (columns 1 and 2), placebo (column 3) and eFSR rating (columns 4 and 5). The time course of mean HbO changes (µM) in each ROI are plotted in those who received THC and were impaired (Col 1), received THC and were unimpaired (Col 2), received placebo THC (Col 3), received an impaired rating from an eFST (Col 4), and in those who received a not-impaired rating from an eFST (Col 5). Pre-dose HbO response is shown in blue, and peak dose (Scan 2 or 3, whichever had the higher intoxication rating) is shown in red. Blue and red lines represent the mean and the standard error of the mean in each group. Yellow lines represent single timepoints in which the group differences between pre and peak HbO were significant (using a paired t-test with Benjamini-Hochberg FDR correction and *p* < 0.05). For statistical purposes, only matched pre and post-dose scans were included, which resulted in a sample size of 77 (out of 80) concordant impaired subjects and 55 (out of 57) concordant not clearly impaired subjects. MPFC middle prefrontal cortex, RDLPFC right dorsolateral prefrontal, RVLPFC right ventrolateral prefrontal cortex, LDLPFC left dorsolateral prefrontal cortex, and LVLPFC left ventrolateral prefrontal cortex.

HbO response during the n-back task pre- and post-THC and placebo in the five ROIs assessed are shown in Fig. 3. Participants operationalized as impaired with concordant impaired CCR and algorithm ratings had increased HbO following THC in all five ROIs queried. There was no significant change in HbO concentration during the n-back task in any ROI in participants with concordant ratings as not clearly impaired following a similar dose of THC or in participants following placebo. Participants with self-reported high >50 of 100 on the DEQ also had significantly increased HbO concentration in all PFC regions post-THC, while those with self-reported high <50 had no significant HbO change post-THC (Supplementary Fig. 3). In participants in whom the DRE-administered eFST indicated impairment, fNIRS signal followed a similar pattern, but with significantly increased HbO concentration post-THC only in the MPFC and for a brief 3.6-s interval in the time course of the right DLPFC (Fig. 3, right 2 columns).

### Individual level classification of impairment with machine learning algorithms using only brain data, using operationalized determination of impairment by clinical consensus and HR/self-rating algorithm as ground truth

**Fig. 4:**
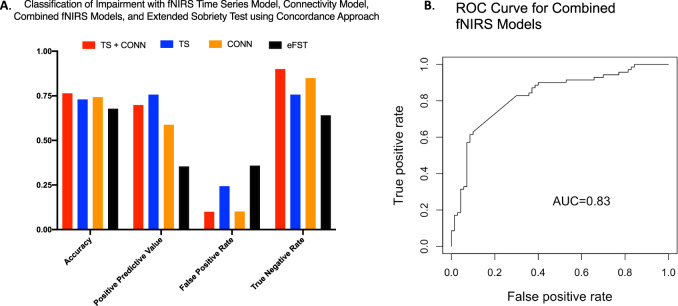
Machine learning metrics. **A** TS, time series, CONN, connectivity, eFST, extended field sobriety test. **B** Receiver operative characteristic (ROC) curve for combined fNIRS models.

Impairment classification using pre- and post-dose fNIRS scans with ensemble learning with fNIRS time series and connectivity data, using fNIRS scans with concordant consensus clinical rating and HR/self-rated high algorithm impairment determination as ground truth, yielded accuracy of 76.4%, a positive predictive value (PPV) of 69.8%, and a FPR of 10.0% (Fig. 4A; Supplementary Table 2), and an area under the receiver operating characteristic (ROC) curve of 0.83 (Fig. 4B). Impairment classification using only features from the fNIRS time series data classified impairment with accuracy of 73.0% and a PPV of 75.7%. Using only fNIRS connectivity data resulted in accuracy of 74.3% and a PPV of 58.8%. Impairment classification using eFST evaluation without fNIRS features, with concordant clinical and algorithmic impairment ratings as ground truth, yielded accuracy of 67.8%, and a PPV of 35.4%, with a FPR of 35.9%. A two-sample test of proportions found that the FPR for the eFST evaluation of 35.9% was significantly higher (*p* < 0.001) than the 10% rate from the ML algorithm. Importantly, the ML classifier built using only data from the post-THC scans (no pre-dosing normative data), performed with a PPV of 72.6%, accuracy of 77.3%, and a FPR of 17.9% (Supplementary Table 3).

### Using “Not Clearly Impaired” scans as a test (hold-out) set, machine learning algorithms accurately classified these scans

Scans from the 57 participants who were given THC but operationalized as not clearly impaired by concordant consensus clinical rating and HR/self-rated high algorithm were used as a test dataset, using the above classifier. With ensemble learning with fNIRS time series and connectivity data, this test dataset yielded an accuracy of 84.6%, and a FPR of 15.4%. With time series data only (no connectivity), this test yielded an accuracy of 89.2%, and a FPR of 10.8%.

### Adverse events

All adverse events were considered mild to moderate and were transient. Asymptomatic severe hypertension (SBP > 180) was observed in 2 participants, correlating with peak drug effect; see Supplement.

## Discussion

This study replicates literature from fMRI [15], PET [31], EEG [16], and ASL [17] studies showing increased activation of the PFC after THC exposure, and extends this literature by showing that such activation is specific to the state of acute THC impairment. Further, we demonstrate that standard ML methods, using PFC fNIRS measurements during a simple memory task, can distinguish individual participants who are impaired due to THC intoxication from those who are not clearly impaired or only mildly intoxicated, with high accuracy.

At the group level, increased PFC HbO response was observed in participants considered impaired from THC intoxication with concordant clinical consensus and algorithmic approaches and not in those rated not clearly impaired by this method following THC or placebo. PFC HbO response similarly distinguished participants who rated themselves as feeling quite intoxicated from those rating themselves less high following THC and following placebo. These findings suggest PFC activation is a marker for acute THC intoxication itself, rather than for recent exposure of a similar dose of THC that did not result in intoxication or of chronic THC exposure alone. This HbO response alone was used to build an impairment classifier that performed with higher accuracy, 76.4% vs 67.8%, and, importantly, significantly lower FPR, 10.0% vs 35.9%, for impairment than the DRE-administered eFST.

Increased HbO response, observed across all PFC regions in participants impaired following oral THC, may be caused by reduced brain efficiency during acute THC intoxication, such that greater effort is required to complete the simple 2-back working memory task. This pattern is reported in people with schizophrenia and their siblings during working memory tasks and interpreted to represent increased neural effort for normative performance [32, 33]. That we observed increased HbO across the PFC sub-regions, suggests this effect is driven by a global, task-based increase in HbO in the PFC.

fNIRS time series features have been used in ML classifiers of mild cognitive impairment that perform with reported 83% accuracy [20], suggesting that an fNIRS imaging approach, using time-course features, may detect biomarkers for such clinical states such as dementia and intoxication. We further showed, using a recurrent neural net that explored dynamic connectivity during the n-back task, that an entirely different ML approach could also accurately classify those who were impaired from THC from those who were not clearly impaired following THC and placebo. Dynamic connectivity is a relatively new method of assessing temporally correlated activation states of discrete brain regions over time [34], assessing variability in the strength and spatial organization of functional connectivity across brain regions, here across regions of the PFC. Dynamic connectivity is associated with vigilance [35], arousal [36], and emotional state [34]. Thus, it is not surprising that PFC dynamic connectivity was different in those who were impaired from those who were not clearly impaired following similar THC doses.

There are limitations to this study. Whereas most acute cannabis administration trials use fixed dosing and examine group effects [3, 37, 38], here we used individually tailored dosing to account for the wide variability in tolerance to cannabis, while aiming to achieve impairment due to intoxication in all participants while minimizing adverse experiences for participants. Future studies could develop pharmacokinetic models for individualized oral cannabis administration that would result in a target exposure, as has been done with alcohol [39] or use vaped cannabis which would allow participants to self-titrate their dose to intoxication. Even with flexible dosing, only approximately half of participants achieved such significant intoxication that we were confident that performance in such activities as driving would be impaired. It is worth noting that participants who, even with individualized dosing, did not experience significant intoxication with active study drug were generally heavier users, as suggested by the difference in urine THCCOOH concentration between the impaired and not clearly impaired groups (Table 1). This underscores the idea that for many heavier users who have developed marked tolerance to impairing effects of THC, a low legal cutoff for THC in saliva or blood may not capture impairment as intended.

Second, assessing impairment is challenging, as there is no accepted objective definition of impairment. In this trial, we tried to differentiate mild intoxication that *may not* impair performance (which was seen in nearly all participants) from impairment due to significant intoxication that would almost certainly result in compromised driving ability. We assessed impairment via physiologic signs (heart rate change), self-reported ‘high,’ clinical assessment by study staff, nurses, and physicians who interacted with the participants and the eFST by a trained evaluator. While the eFST classified 21.6% of participants as impaired following placebo dosing, chronic cannabis exposure has been associated with deficits in neurocognitive performance [40]; these may be mistaken for effects of acute intoxication on the eFST.

Third, since this trial began, effects of cannabis have been shown on with the Digit Symbol Substitution Task (DSST), Divided Attention Task (DAT), and Paced Auditory Serial Addition Task (PASAT) [37, 38, 41–43], as well as the DRUID Smartphone/Tablet Application [44]. Future trials could incorporate these validated tasks into assessments of impairment. The n-back task was chosen here because it is widely used in fNIRS as well as fMRI to reliably activate the PFC [33]. While those who were impaired from THC demonstrated expected decrements in n-back task performance, this task was not intended to be an objective measure of impairment.

Fourth, we did not collect biological samples before and after administration of study drug on study days for THC assay that would have allowed for objective comparison of THC exposure in addition to dronabinol dose. Although there is an extensive literature describing poor correlation between blood and saliva THC levels and impairment [1, 37, 38], without such bioassays, this trial cannot independently replicate those findings or understand how the fNIRS method compares to per se concentration cutoffs being used in some places as the legal standard for driving. Further, we cannot verify that no participant had THC in their system prior to dosing, which would have been detected with blood or saliva THC concentrations pre-dosing.

Finally, we did not ascertain whether change in PFC activation ascertained with fNIRS with impairment is specific to THC impairment due to THC intoxication. Studies underway will assess the impact of alcohol, sleep deprivation, and other sources of impairment on this fNIRS signature.

Here we report feasibility of fNIRS as a potential method as assess impairment from cannabis. fNIRS has several characteristics that make it suitable for real-world utility, such as roadside application. There are now portable, lightweight, wireless, battery-powered fNIRS devices that allow data to either be stored on wearable recording units or transmitted wirelessly to a laptop [45]. fNIRS can be performed without sedation, and while a participant is moving, making it suitable for use in real-world settings across the lifespan [46]. Finally, set-up time for fNIRS is minimal compared to other portable imaging modalities such as EEG measurements, particularly when using optodes only on the forehead, obviating the need to adjust optodes to get a good signal in the presence of hair [47]. Indeed, fNIRS experiments are increasingly performed outside the laboratory and in everyday life situations [48–50].

Challenges to fNIRS use in the field exist. Although fNIRS is quite tolerant to movement, specific movements like raising of eyebrows cause significant motion artifacts. In outdoor environments, optical detectors must be shielded from sunlight which can saturate detectors [49]. Further, physiological confounding of fNIRS signals by cardiovascular and respiratory function may be a significant issue if used in a law enforcement environment where people may be anxious. Short-separation channels, created by placing a light source close to a detector to record data from extracerebral tissue, can identify physiological and hemodynamic signals. Such extracerebral signal components (e.g., superficial skin blood flow) can then be removed to isolate brain signal [51]. The most significant barrier to the use of fNIRS as a real-world tool for detecting impairment is not likely to be limitations of technology, but rather the complexity of physiology, whereby blood flow in the PFC may be influenced by factors such as other medications, neurological/ psychological comorbidities, or a combination of these factors. Thus, in the field, fNIRS measurements may be most useful in conjunction with saliva, breath, or urine bioassays showing presence of the drug, and fNIRS assessment showing probable impairment from the drug. Even so, this may present a significant advantage compared with either oral fluid THC tests, which only assess the presence of the cannabis, not impairment, or with DRE evaluations, which are resource-intensive, time-consuming, and have been reported to be subject to bias [8].

In summary, impairment due to THC intoxication was associated with increased PFC activation on a simple memory task assessed with fNIRS. These measures alone classified participants as impaired vs exposed but not clearly impaired with high PPV and accuracy. Combining time course and connectivity methods of assessing brain activation improved impairment detection. As we showed that there was no difference in THC dose between those who became impaired from those who did not following THC, it is likely that a brain- or behavior-based metric (e.g. eye tracking or cognitive testing [52]), rather than a per se blood or oral fluid limit of THC, is required to distinguish THC impairment from simple exposure [1]. Future work is warranted to determine if observed brain signatures are specific to THC intoxication-related impairment or are a more general signature of impairment.

## Supplementary information

Supplementary Information for Identification of ∆9-tetrahydrocannabinol (THC) Impairment Using Functional Brain Imaging

## Data Availability

All data, code, and materials used in the analyses can be provided by Jodi Gilman and Massachusetts General Hospital pending scientific review and a completed data use agreement/material transfer agreement. Requests for all materials should be submitted to Jodi Gilman.
