# Supplementary material for: Identification of ∆9-tetrahydrocannabinol (THC) impairment using functional brain imaging
Source: Neuropsychopharmacology. 2022 Jan 8;47(4):944–52. doi: 10.1038/s41386-021-01259-0 (PMC8882180; doi:10.1038/s41386-021-01259-0)
Supplement: Supplementary file 1 — Supplementary Information for Identification of ∆9-tetrahydrocannabinol (THC) Impairment Using Functional Brain Imaging [file 41386_2021_1259_MOESM1_ESM.docx]

**Supplementary Information for**

Identification of ∆9-tetrahydrocannabinol (THC) Impairment Using Functional Brain Imaging.

Jodi M. Gilman, PhD^1,2,3^* William A. Schmitt, AB^1,3^ Kevin Potter, PhD^1,2^ Brian Kendzior, BS, Gladys N. Pachas, MD^1,2^ Sarah Hickey, RN^1^ Meena Makary, PhD^3,4^ Marilyn A. Huestis, PhD ^5^ A. Eden Evins, MD, MPH^1,2^

^1^Massachusetts General Hospital (MGH) Department of Psychiatry, Boston, MA, USA

^2^Harvard Medical School, Boston, MA, USA

^3^MGH/HST Athinoula A. Martinos Center for Biomedical Imaging, Department of Radiology, Massachusetts General Hospital, Harvard Medical School, Charlestown, MA, USA

^4^Faculty of Engineering, Cairo University, Cairo, Egypt

^5^Institute of Emerging Health Professions, Thomas Jefferson University, Philadelphia, PA, USA

*Corresponding author: Jodi M. Gilman, PhD, MGH Center for Addiction Medicine, 101 Merrimac St, Suite 320, Boston, MA 02114; Tel: 617-643-7293; Fax: 617-643-1998; Email: [jgilman1@mgh.harvard.edu](mailto:jgilman1@mgh.harvard.edu).

**Supplemental Methods**

***THC Dosing***

To determine a clinically tailored dose of THC that would maximize the chance for intoxication while minimizing adverse events such as anxiety, panic, paranoia, and hemodynamic change, clinical staff (SH, GP) took a careful history of cannabis product use, including age of onset and quantity/dose, frequency, and route of cannabinoid use over time, with particular attention to quantity and frequency of recent use, 0-100 rating of subjective intoxication with usual use, recent oral cannabinoid dosing, if any, with self-rated intoxication and adverse events. Study physicians (GP and AEE) determined during the screening visit the dronabinol dose most likely to be both well-tolerated and to produce intoxication, up to a maximum dose of 80 mg. Primary dosing considerations were degree of expected tolerance and prior adverse experiences with THC-containing products, particularly edibles. Doses were calculated for each participant individually based on their reported average cannabis frequency and demographic factors such as age and sex (9). Participants with a systolic blood pressure >130 and/or diastolic blood pressure >90 were given either 5mg (unlikely to produce further blood pressure increase) or their calculated dose based on the recommendation of the study physician.

We developed a dosing method using demographic variables (sex, race, and BMI), and indicators of THC tolerance (age of first use, amount in grams per day in the past 6 months, number of THC sessions per day, number of THC days/week, a composite measure of ‘rate’, equal to the average amount used per time (in grams) divided by the average level of intoxication achieved with that amount (on a scale from 0-100), largest amount used (in grams) in one sitting in the previous year, a scale (0-100) describing level of intoxication (“high”) achieved with that amount, use of ingestible THC, largest amount ingested (in milligrams) in one sitting in the previous year, history of an adverse event with THC (includes paranoia, fainting, nausea, vomiting, dizziness), and a positive family history of hypertension. Each of these was assigned a value to create a dosing “score” and that score was converted to a THC dose; indicators of tolerance increased the score, and indicators of adverse events lowered the score. This dosing was used as a guideline for the physicians. See below for an example subject:

***Example Dosing Table***

| 1 | Sex | Female | 0 |  | | 5 |
| --- | --- | --- | --- | --- | --- | --- |
|  |  | Male | 5 |  | |  |
| 2 | Race | white | 1 |  | | 1 |
|  |  | non white | 0 |  | |  |
| 3 | When did you start using cannabis regularly? | <=16 yrs | 1 |  | | 1 |
|  |  | >16 yrs | 0 |  | |  |
| 4 | How many months ago did you become a regular cannabis user? | <6 months | 0 |  | | 2 |
|  | (at least once every week) | 6 to 12 months | 1 |  | |  |
|  |  | >12 months | 2 |  | |  |
| 5 | How many grams per day did you smoke in the past year | <0.5 gr | 0 |  | | 1 |
|  |  | 0.5-1 gr | 1 |  | |  |
|  | if use change in the past 6 months, consider the heaviest use | 1.1-1.9 gr | 2 |  | |  |
|  |  | 2-2.9 gr | 3 |  | |  |
|  |  | 3-3.9 gr | 4 |  | |  |
|  |  | >=4 gr | 5 |  | |  |
| 6 | How many times a day do you use cannabis? | 1 | 0 |  | | 2 |
|  |  | 2 | 1 |  | |  |
|  |  | 3 | 2 |  | |  |
|  |  | 4 | 3 |  | |  |
|  |  | 5 or more | 4 |  | |  |
| 7 | How many times a week do you use cannabis? | 1 to2 | 0 |  | | 2 |
|  |  | 3 to 4 | 1 |  | |  |
|  |  | 5 to 6 | 2 |  | |  |
|  |  | everyday | 3 |  | |  |
| 8 | Per amount used in mg (see Q5), on a scale from 0-100, how high did you get on average?   - *this is only used to create a score, see at the bottom of the table)* |  |  |  | |  |
|  | amount (mg) per time/high score (1-100) |  |  |  | |  |
| 9 | What is the most cannabis, in grams, you have ever used in a setting in the past year? | <1 gr | 0 |  | | 1 |
|  |  | 1-1.9 gr | 1 |  | |  |
|  |  | 2-2.9 gr | 2 |  | |  |
|  |  | 3-3.9 gr | 3 |  | |  |
|  |  | 4-4.9 gr | 4 |  | |  |
|  |  | 5 or more | 5 |  | |  |
| 10 | On a scale from 0-100, how high did you get? | <50 | 2 |  | | 1 |
|  |  | 50 to 70 | 1 |  | |  |
|  |  | 80 to 100 | 0 |  | |  |
|  |  |  |  |  | |  |
| 11 | Have you ever used cannabis edibles? | yes | 1 |  | | 1 |
|  |  | no | 0 |  | |  |
| 12 | What is the most you have ever had in edibles in one occasion? | <20 mg | 0 |  | | 1 |
|  |  | 20-30 mg | 1 |  | |  |
|  |  | 31-40 mg | 2 |  | |  |
|  |  | 41-60 mg | 3 |  | |  |
|  |  | 61-70 mg | 4 |  | |  |
|  |  | 71-80 mg | 5 |  | |  |
|  |  | 81-90 mg | 6 |  | |  |
|  |  | >91 mg | 7 |  | |  |
|  |  |  |  |  | |  |
| 13 | On a scale from 0-100, how high did you get from those edibles? | <50 | 2 |  | | 1 |
|  |  | 50 to 70 | 1 |  | |  |
|  |  | 80 to 100 | 0 |  | |  |
| 14 | Have you ever had a bad high (includes paranoia, fainting, moderate/severe nausea, vomiting, dizziness)? | yes | 0 |  | | 0 |
|  |  | no | 1 |  | |  |
| 15 | Do you have a medical history/family medical history of hypertension? | yes | 0 |  | | 0 |
|  |  | no | 1 |  | |  |
| 16 | BMI | <=21 | 0 |  | | 0 |
|  |  | 22-25 | 1 |  | |  |
|  |  | 26-35 | 2 |  | |  |
|  |  | >35 | 3 |  | |  |
|  |  |  |  | **Summed Score** | **19** | |
|  |  |  |  | Summed Score x 2 | 38 | |
|  |  |  |  | Mg of THC / subjective high (e.g. 500/100) | 5 | |
|  |  |  |  | **DOSE (mg)** | **43** | |

***Blinding of Study Medication.***

The MGH research pharmacy generated a blinded randomization code for order of dosing and dispensed blinded drug in the dose ordered and identical placebo for use on separate study days. All participants, study staff, assessors, and DREs were blinded.

***Defining Impairment:*** We developed a two-stage process for operationalizing ground truth impairment for the purpose of identifying fNIRS scans to be used in building the ML models. Post-dose scans were considered to be impaired if they were conducted when participants were rated as impaired by two clinical raters, using all but fNIRS data, in participants who were also identified by an algorithm using physiologic (heart rate) and psychologic (self-rating of intoxication) inputs to discriminate THC from placebo with a low false positive rate.

*A* *consensus clinical assessment* (JG, GP, AEE) was developed to identify participants with clinical signs of impairment due to intoxication. Two raters (JG and AEE) considered the following data for both THC and placebo study visits: serial ratings of self-reported high and heart rate during study visits, blinded study staff rating of impairment (yes/no to the single question: would you ride in a car with this person driving at their peak high during the study visit?), blinded DRE eFST determination of impairment, driving simulator data when available, dose of study medication, and n-back task performance and independently generated a clinical rating for each fNIRS scan based on these variables, blind to fNIRS data, on a scale of 0-100%, indicating the clinical likelihood the participant was impaired due to THC intoxication when a post THC scan was conducted. Scans for which both raters scored 75 or greater were considered impaired scans by these clinical consensus ratings (CCR).

To reduce risk for bias with clinical ratings, we developed an *Algorithmic Method for Determining Intoxication* that was used as a second criterion required to consider a scan to be ground truth impaired for building the ML classifier. This algorithm was based on physiologic (heart rate) and psychologic (self-rating of intoxication (‘high’) on the DEQ) indicators of drug effect only, with active THC or placebo dosing as ground truth. Both HR and self-reported high were collected several times before and after each scan; we used linear interpolation to estimate heart rate and self-reported high at the time of each post-dose scan. Change in heart rate was then computed as the difference between the average pre-dose value and the interpolated value at the time of a specified post-dose scan. We then sought to establish a set of cutoff values for heart rate change and self-reported high that we could use to label people as intoxicated or not intoxicated at the time of the scan, using placebo or THC dosing as ground truth. A scan was predicted to be post-THC if the observed change in heart rate **or** the observed self-reported high was equal to or greater than the associated cutoff; otherwise, the scan was predicted to be post-placebo. Cutoffs were chosen based on values that produced the highest d' score for correctly classifying post-THC or post-placebo scans. The measure d' was used as the metric to optimize cutoffs in order to both maximize hit rates and minimize false alarm rates.

We used a 10-fold cross-validation method, in which participants were randomly split into 10 groups, and the data for subjects in a given fold were reserved as a validation sample, while the data from the other nine folds were combined into a single training sample. The training sample was then used to identify the optimal pair of cutoffs via a grid search approach. Associated d’ scores for all possible cutoffs (ranging from 1 to 70 bpm for change in heart rate, and 1 to 100 for self-reported high) were computed and the pair of cutoffs associated with the highest d’ were selected. Predictive performance (e.g., hit and false alarm rates and associated d’ scores), was computed using the withheld validation sample. This process was repeated so that data from each fold served as a validation sample, resulting in 10 sets of cutoffs and associated metrics of predictive performance. Finally, the optimal pair of cutoffs and the associated predictive performance were computed by averaging over the 10 sets: averaged cutoffs found that a participant was classified as 'intoxicated' if either (1) self-reported high from the DEQ > 59, or (2) change in heart rate (from mean pre-drug to the time point closest to the second fNIRS scan) > 19 beats per minute. The 10-fold cross-validation results indicate that these cutoffs yielded a hit rate of ~44.3%, false positive rate of ~0.4%, and an overall accuracy of ~72%.

***Analysis of fNIRS Data*:** fNIRS analyses were conducted using Homer2 open source software (MGH-Martinos Center for Biomedical Imaging, Boston, MA), implemented in MATLAB (Mathworks, Natick, MA) (20). The NIRS signal was first converted into optical density, then motion artifacts in the signal were detected and corrected by a hybrid method based on spline interpolation and Savitzky-Golay filtering (21). fNIRS signals were preprocessed with a high-pass filter using cutoff frequencies of 0.01Hz to remove baseline drift and low frequency oscillations, and a 0.5Hz low-pass filter to reduce impact of heartbeat pulsations and high frequency noise. The modified Beer-Lambert law was applied to calculate hemoglobin concentration changes with a partial pathlength factor of 6 (18, 22, 23). To obtain an average response to the N-back task for each of the 20 channels in each participant, the hemodynamic response function (HRF) was estimated by a general linear model (GLM) approach that used ordinary least squares. The HRF was modeled as a series of consecutive Gaussian functions with a standard deviation of 1 sec and their means, separated by 1 sec over the time range of -2 sec to 40 sec (9, 10) as response of the hemodynamic change usually occurs about 5–6s after neuronal activity (24). We then performed our primary statistical analysis on the average over the following time windows for each ROI: ‘pre’ dose scans (e.g. pre-THC, pre-placebo), and ‘peak’ scans at 100 minutes after THC/placebo dosing, or at 200 minutes in several participants who reported late peak intoxication. Differences between pre dose and peak dose scans were analyzed via paired t-tests at each time point in the fNIRS time course. Statistical results were corrected for multiple comparisons using the Benjamini-Hochberg method with a false discovery rate of 0.05. We defined five regions on interest (ROIs) based on channel location. These ROIs are middle prefrontal cortex (MPFC, channels 7, 8, 9, 10, 11, 12, 13, 14); right dorsolateral prefrontal cortex (RDLPFC, channels 15, 17, 18); right ventrolateral prefrontal cortex (RVLPFC, channels 16, 19, 20); left dorsolateral prefrontal cortex (LDLPFC, channels 1, 2, 5); and left ventrolateral prefrontal cortex (LVLPFC, channels 3, 4, 6); See Figure 1A.

***Machine Learning Models:*** Pre-processed data from impaired and placebo scans were used to build two models.

*Temporal Feature Maps from Time-Series Data*: To create temporal feature maps, we extracted 95 numerical values (19 from each of the 5 ROIs): mean HbO values for time segments 0-5s, 5-10s, 10-15s, 15-20s, 20-25s, 25-30s, 30-35s, and 35-40s, slope (5-15s), skewness (0-15s), kurtosis (0-15s), area under the curve (0-15s, 15-40s), time to the first and second extremum of HbO, magnitude of the first and second extremum of HbO, and average and standard deviation of HbO after the second extremum (Figure 1B). These 95 predictive features trained XGBoost (<https://xgboost.readthedocs.io/en/latest/>), an open-source distributed gradient boosting library that is normally used to train gradient-boosted decision trees and other models, to predict impairment. For each participant, we used 95 features from the pre-dose scan paired with the 95 features from the same-day post-dose scans, though we did not specify in the model which scans were pre- and which were post-dose. If there were two impaired post-dose scans, the model randomly selected which scan to use. We then conducted a grid search over XGBoost hyperparameters, which informed hyperparameter selection. In order to minimize false positives, we created a weighted metric function in which we penalized the model for false positives (25).

*Recurrent Neural Network (RNN) Model from Connectivity Data:* To build our RNN model, we computed pairwise correlations (Pearson’s correlation coefficients) between the HbO concentration change values of all possible channel pairs across the 6-minute n-back task. Dynamic connectivity was assessed using a “sliding window” approach (26), in which correlation coefficients of connectivity in regions of interest quantified the time-varying behavior of the these coefficients over the duration of the N-back task. Thus, to investigate dynamic, rather than static, connectivity, we computed connectivity matrices with sliding windows of 300 time points, and a skip of 100 time points, so that each window corresponded to a small segment of the scan. For each window, we computed the instantaneous functional connectivity matrix based on correlating the time course in sliding windows. As in the feature map analysis, for each participant, the dynamic connectivity matrices from the pre-dose scan were paired with the matrices from the same-day post-dose scan(s). We then trained an RNN model architecture with the sliding-window correlation matrix feature vector as the input (Figure 1B). In this RNN, the core component consisted of fully connected layers that mapped the input to a latent representation, which in turn fed to a hidden state with recurrent connections. A probabilistic prediction was computed at every time point by applying a fully connected layer to the hidden state. We used tanh for all nonlinearities and implemented a fully connected neural network with 5 hidden layers and 128, 64, 32, and 16 nodes. The output of this model was a fully connected neural net with a single hidden layer of 64 dimensions and consisted of recurrent connections that captured temporal dynamics. This model estimated the probability of the participant’s impairment. A categorical cross-entropy loss trained the RNNs, and implemented the neural networks in Keras, with a TensorFlow back-end. Grid search with hyper-parameters and alternating direction method of multipliers (ADMM) were employed for optimization. Learning was terminated when validation loss stopped improving.

***Sample Size Determination*** The sample size was pre-specified in our grant application. Since no other studies have attempted to predict impairment using fNIRS measurements, no effect size could be estimated. Sample size was a practical consideration determined by the budget and timeline of the R42 grant and our ML collaborators, who instructed us to collect as many scans as possible for the funds allotted. Formal power analyses are typically framed around p-values, which is not the output of ML analyses.

***Statistical Analysis.***

*Analysis of Cognitive Performance:* To evaluate performance in the 2-back condition of the N-back task, we converted the number of hits (the number of times a participant correctly pressed the response key when a target letter was onscreen), and false alarms (the number of times a participant pressed the response key in the absence of a target letter onscreen) into estimates of signal detection (d’), via the formula: Φ^-1^(proportion hits ) – Φ^-1^ (proportion false alarms), where Φ^-1^ is the inverse of the cumulative distribution function for a Gaussian distribution. Cases of perfect performance were corrected via a log-linear approach.

**Adverse Events.** Thirty-eight participants reported adverse events that were considered related to THC. The most common were anxiety (9 participants), vomiting (7 participants), nausea (6 participants), and dry mouth (5 participants). Dronabinol-induced tachycardia (HR>100) was observed in 41 participants and elevated blood pressure (systolic blood pressure (SBP) > 140) in 30 participants. All adverse events were considered mild to moderate and were transient. Asymptomatic severe hypertension (SBP > 180) was observed in 2 participants, correlating with peak drug effect.

**Consort Diagram**

**
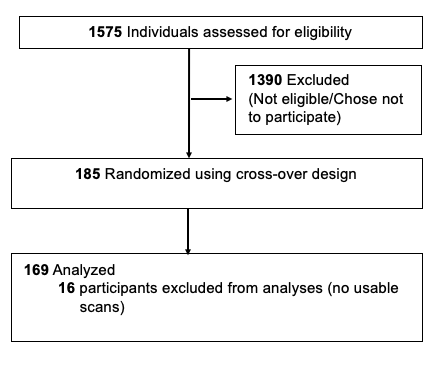
**

**Supplementary Figure 1. Consort diagram of Participant Enrollment**


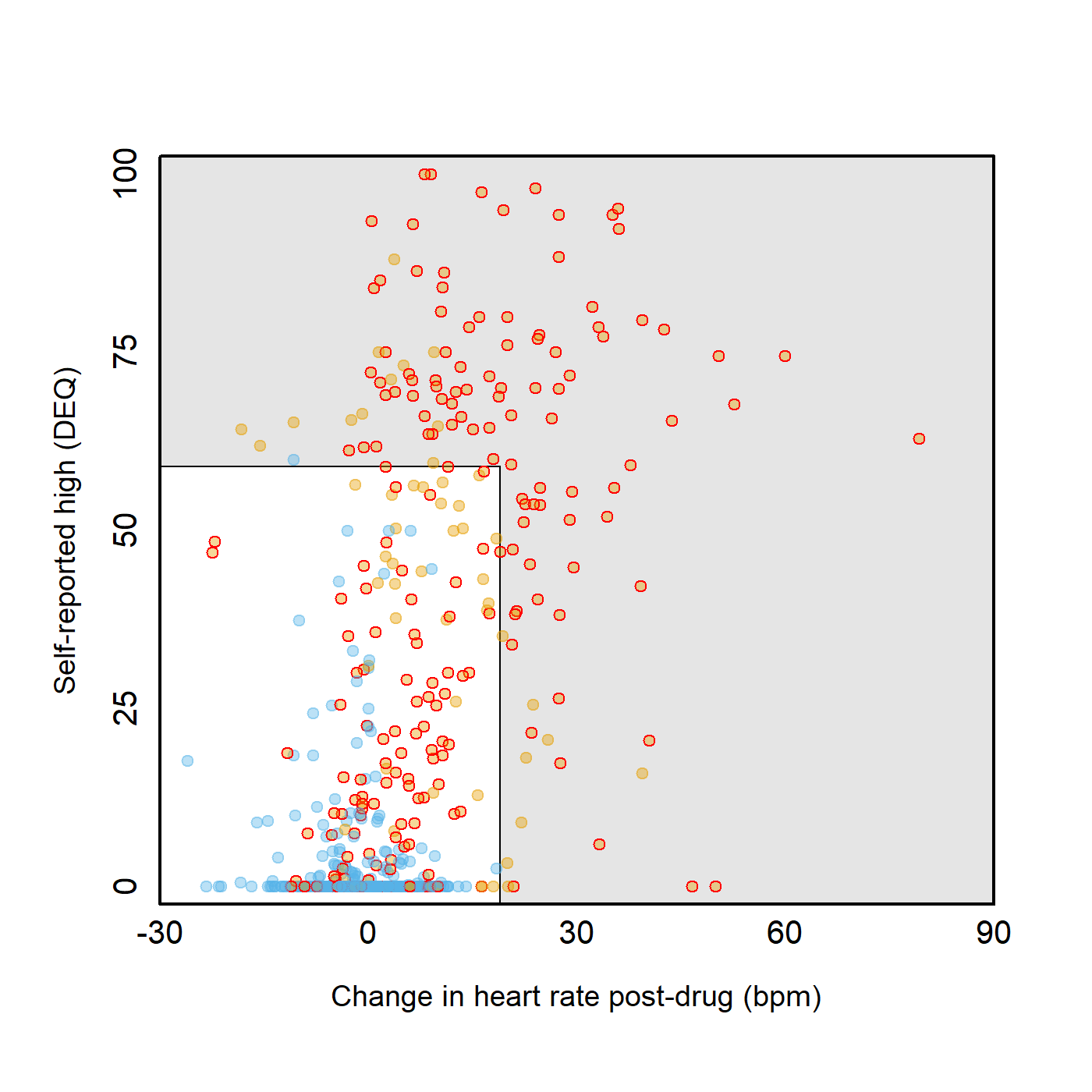


**Performance of Algorithmic Method for Determining Impairment and Concordance with Clinical Ratings**

Intoxicated by Algorithm

Not intoxication by Algorithm

THC, concordant impairment ratings (used in ML)

THC, discordant impairment ratings

Placebo (used in ML)

Supplementary Figure 2. Algorithmic and Clinical Method for Determining Impairment. Using drug or placebo as ground truth, we used two measures of intoxication, change in heart rate (x-axis) and DEQ self-reported ‘high’ (y-axis) to create a grid of cut-offs for self-reported high and change in heart rate. Blue circles indicate post-placebo scans and orange circles indicate post-THC scans. Circles within the grey box were called “not intoxicated” by the algorithm, and any circles outside of the grey box were called “intoxicated.” 10-fold cross-validation results for the algorithm indicated that these cutoffs yielded a hit rate of ~44.3%, false positive rate of ~0.4%, and an overall accuracy of ~72%. Expert raters (AEE and JG) also created a clinical consensus rating of impairment for each participant at each scan using measures not available to the algorithm (e.g. blinded study staff ratings, DRE evaluations, comparison of self-reported high after blinded placebo dosing with post-THC self-report). There was an 75.5% match between the algorithmic and clinical methods for defining impairment.


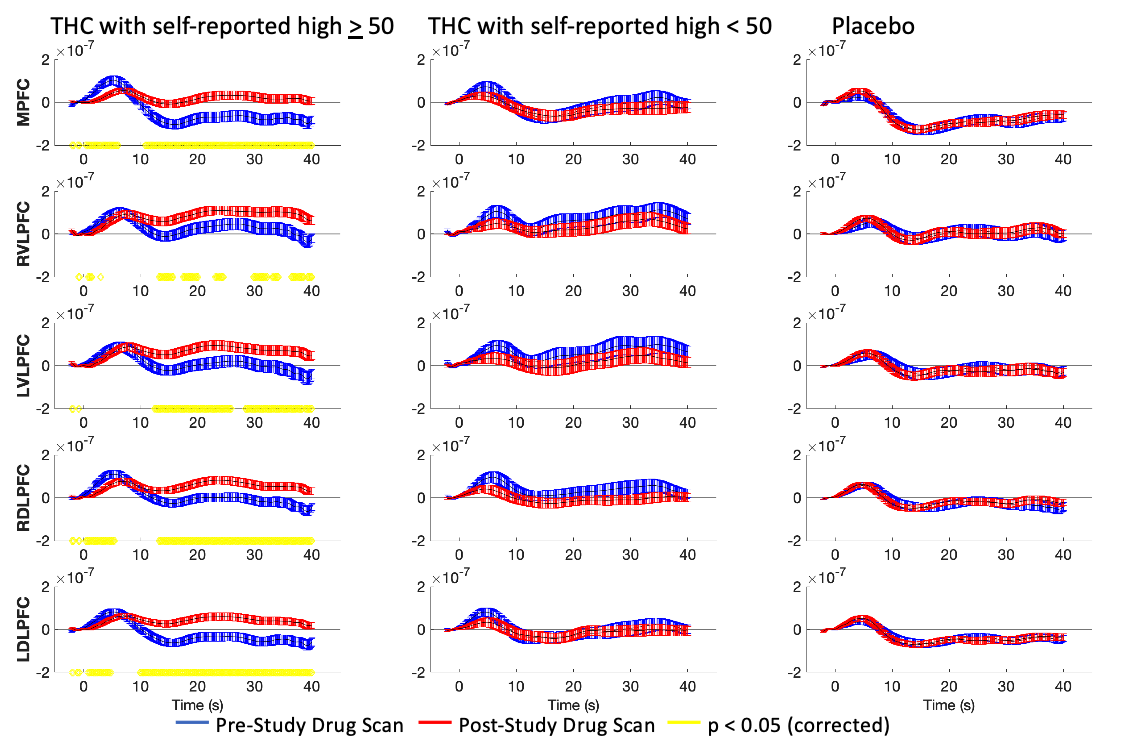


Supplementary Figure 3. fNIRS signal during n-back time series in those who reported becoming high from THC vs. those who did not and placebo. The time course of mean HbO changes (µM) in each ROI are plotted in those who received THC and had an interpolated self-reported intoxication of >50 at the time of the scan (left), received THC and had an interpolated self-reported intoxication of <50 intoxication (middle) and in those who received placebo THC (right). Pre-dose HbO response is shown in blue, and post dose is shown in red. Yellow lines represent single timepoints in which the group differences between pre and peak HbO were significant (using a paired t-test with Benjamini-Hochberg FDR correction and p<0.05). Error bars illustrate the standard error of the mean HRF across subjects.

**Supplementary Table 1.** **Effects of Study Medication in those who were and were not considered to have ground truth impairment rated as impaired with concordant ratings (algorithm and clinical consensus) following THC.**

| Measure | Time point | Rated Impaired Post-THC^1^ | Rated Not Clearly Impaired Post-THC^1^ | T-test statistic | p-value |
| --- | --- | --- | --- | --- | --- |
|  |  | (N=80) | (N=57) | (df) |  |
| THC dose |  | 35.6 ± 11.5 | 34.8 ± 16.1 | 0.30 (95.3) | p = 0.77 |
| 'Feeling high'  DEQ | Peak | 78.1 ± 17.9 | 38.3 ± 30.6 | 8.81 (82.9) | p < 0.001 |
|  | Scan used in ML | 54.2 ± 19.4 | 18.6 ± 17.2 | 11.31 (128.4) | p < 0.001 |
|  | Post-dose scan 1 | 55.7 ± 29.8 | 18.7 ± 21.5 | 8.44 (135) | p < 0.001 |
|  | Post-dose scan 2 | 50.0 ± 25.4 | 19.3 ± 20.5 | 7.43 (120.4) | p < 0.001 |
| Heart rate  Change from  pre-baseline avg.  (bpm) | Peak | 31.8 ± 17.1 | 12.7 ± 10.6 | 8.06 (132.8) | p < 0.001 |
|  | Scan used in ML | 17.8 ± 12.3 | 2.6 ± 6.6 | 9.3 (126.3) | p < 0.001 |
|  | Post-dose scan 1 | 16.8 ± 16.5 | 2.2 ± 7.3 | 7.03 (116.4) | p < 0.001 |
|  | Post-dose scan 2 | 18.8 ± 11.4 | 3.5 ± 6.6 | 9.34 (114.2) | p < 0.001 |
| N-back d' score (Post-dose minus pre-dose) |  | -0.22 ± 0.75 | 0.21 ± 0.57 | 3.70 (130.7) | p < 0.001 |
|  |  |  |  |  |  |
|  |  |  |  |  |  |

^1^ Those rated as impaired and not impaired/unsure were rated as such by both Consensus Clinical Rating and HR/Self-rated Intoxication Algorithm.

**Supplementary Table 2. Machine Learning Metrics using Algorithmic, Clinical, and Concordant Definition of Impairment.**

| Model | ACC | PPV | FPR | TPR | FNR | TNR |
| --- | --- | --- | --- | --- | --- | --- |
| *Algorithmic Definition of Impairment* | | | | | | |
| TS + CONN | 0.733 | 0.757 | 0.286 | 0.752 | 0.248 | 0.714 |
| TS | 0.754 | 0.598 | 0.090 | 0.910 | 0.402 | 0.875 |
| CONN | 0.813 | 0.858 | 0.124 | 0.751 | 0.249 | 0.876 |
| eFST | 0.676 | 0.399 | 0.348 | 0.752 | 0.278 | 0.652 |
| *Clinical Definition of Impairment* | | | | | | |
| TS + CONN | 0.800 | 0.858 | 0.130 | 0.730 | 0.270 | 0.870 |
| TS | 0.744 | 0.757 | 0.228 | 0.716 | 0.284 | 0.772 |
| CONN | 0.758 | 0.663 | 0.147 | 0.853 | 0.337 | 0.826 |
| eFST | 0.819 | 0.695 | 0.199 | 0.852 | 0.148 | 0.801 |
| *Concordant (Algorithm + Clinical) Definition of Impairment* | | | | | | |
| TS + CONN | 0.764 | 0.698 | 0.100 | 0.629 | 0.371 | 0.900 |
| TS | 0.730 | 0.757 | 0.243 | 0.703 | 0.297 | 0.757 |
| CONN | 0.743 | 0.588 | 0.102 | 0.898 | 0.413 | 0.850 |
| eFST | 0.678 | 0.354 | 0.359 | 0.833 | 0.167 | 0.641 |

ACC, accuracy; PPV, positive predictive value; FPR, false positive rate; TPR, true positive rate; FNR, false negative rate; TNR, true negative rate; TS, time series; CONN, connectivity; eFST, extended field sobriety test

Supplementary Table 3. Machine Learning Metrics using Algorithmic, Clinical, and Concordant Definition of Impairment Using Only a Single Post Dose fNIRS Scan.

| Model | ACC | PPV | FPR | TPR | FNR | TNR |
| --- | --- | --- | --- | --- | --- | --- |
| *Algorithmic Definition of Impairment* | | | | | | |
| TS + CONN | 0.704 | 0.671 | 0.264 | 0.671 | 0.329 | 0.736 |
| TS | 0.714 | 0.650 | 0.222 | 0.650 | 0.350 | 0.778 |
| CONN | 0.717 | 0.531 | 0.097 | 0.903 | 0.469 | 0.852 |
| eFST | 0.676 | 0.399 | 0.348 | 0.752 | 0.248 | 0.652 |
| *Clinical Definition of Impairment* | | | | | | |
| TS + CONN | 0.779 | 0.758 | 0.200 | 0.758 | 0.242 | 0.800 |
| TS | 0.684 | 0.621 | 0.253 | 0.621 | 0.379 | 0.747 |
| CONN | 0.753 | 0.663 | 0.158 | 0.842 | 0.337 | 0.820 |
| eFST | 0.819 | 0.695 | 0.199 | 0.852 | 0.148 | 0.801 |
| *Concordant (Algorithm + Clinical) Definition of Impairment* | | | | | | |
| TS + CONN | 0.773 | 0.726 | 0.179 | 0.726 | 0.274 | 0.821 |
| TS | 0.770 | 0.740 | 0.199 | 0.740 | 0.260 | 0.801 |
| CONN | 0.722 | 0.603 | 0.160 | 0.840 | 0.397 | 0.814 |
| eFST | 0.678 | 0.354 | 0.359 | 0.833 | 0.167 | 0.641 |

ACC, accuracy; PPV, positive predictive value; FPR, false positive rate; TPR, true positive rate; FNR, false negative rate; TNR, true negative rate; TS, time series; CONN, connectivity; eFST, extended field sobriety test
